# The lived experience of withdrawal from Selective Serotonin Reuptake Inhibitor (SSRI) antidepressants: A qualitative interview study

**DOI:** 10.1111/hex.13966

**Published:** 2024-01-09

**Authors:** Raqeeb Mahmood, Vuokko Wallace, Nicola Wiles, David Kessler, Katherine S. Button, Graeme Fairchild

**Affiliations:** ^1^ Department of Psychology University of Bath Bath UK; ^2^ Bristol Medical School, Centre for Academic Mental Health, Population Health Sciences University of Bristol Bristol UK

**Keywords:** antidepressant, antidepressant withdrawal, depression, discontinuation, primary care, qualitative research

## Abstract

**Background:**

Our knowledge of the broader impacts of antidepressant withdrawal, beyond physical side effects, is limited. Further research is needed to investigate the lived experiences of withdrawal, to aid clinicians on how to guide patients through the process.

**Aim:**

To explore antidepressant users’ experiences and views on the withdrawal process and how it affected their quality of life across multiple life domains.

**Design and Setting:**

We conducted in‐depth qualitative interviews with 20 individuals from the community who had attempted to withdraw from Serotonin Reuptake Inhibitor antidepressants in the past year.

**Method:**

Semi‐structured interviews were conducted online. A topic guide was used to ensure consistency across interviews. The interviews were audio‐recorded and transcribed verbatim and analysed using inductive reflexive thematic analysis.

**Results:**

Five themes were generated. The first highlighted the challenges of managing the release from emotional blunting and cognitive suppression following antidepressant discontinuation. The second related to the negative impact of withdrawal on close relationships and social interactions. The third showed that concurrent with negative physical symptoms, there was a positive impact on health (exercise was reported by some as a coping mechanism). The fourth theme focused on support from GPs and families, emphasising the importance of mental health literacy in others. The final theme underscored the importance of gradual and flexible tapering in enabling a manageable withdrawal experience, and the consideration of timing.

**Conclusion:**

The lived experience of withdrawal significantly impacts individuals’ well‐being. Participants emphasised that withdrawal is not just about physical side effects but also affects their emotional, cognitive, and social functioning.

**Patient and Public Involvement (PPI):**

Eight people attended individual online meetings to share their experiences of antidepressant withdrawal to help inform the study design and recruitment strategy. Insights from these meetings informed the development of the topic guide. Questions about GP involvement, family relationships, and mood and thinking changes were included based on this PPI work. This ensured the inclusion of topics important to antidepressant users and facilitated the researcher's questioning during the interviews.

## 1 INTRODUCTION

Depression is a common and costly mental health condition in the United Kingdom with over 80 million prescriptions for antidepressants, such as Selective Serotonin Reuptake Inhibitors (SSRIs), in 2021/2022 alone.^1^ Many people remain on antidepressants for a long time as evident in the large number of repeat prescriptions.^2^, ^3^ However, there is evidence that between 30–50% of these individuals may not need to take antidepressants in the long‐term.^4^, ^5^ To help patients come off their antidepressants safely, clinicians advise going through a tapering process, reducing the dose over a period of weeks or months.^6^, ^7^ However, the high rates of repeat prescriptions and depressive relapse suggest that antidepressant withdrawal is complex and challenging.^8^ Previous studies of antidepressant withdrawal have largely focused on physical withdrawal symptoms^9^, ^10^ and barriers to withdrawal.^11^, ^12^

Withdrawal effects are experienced by more than half of individuals attempting to discontinue antidepressants: some are mild and short‐term while others are severe and long‐lasting.^10^, ^13^ A systematic review found that 46% of individuals undergoing withdrawal from antidepressants rated their symptoms as extremely severe, with a considerable number of people experiencing these symptoms for more than two weeks, and in some cases several months.^10^ Consequently, concerns about these withdrawal effects and the risk of relapse often lead patients to remain on medication after remission.^14^  A survey of UK GPs found inconsistencies and knowledge gaps regarding withdrawal effects; 68% desired more training in this area.^15^ Distinguishing withdrawal symptoms from early signs of depressive relapse is also challenging.^16^ Qualitative research has shown that patients feel that the antidepressant withdrawal process is misunderstood and not properly managed by their GPs.^17^  Patients report that they have not been well informed about antidepressant tapering and the risks involved in discontinuation and are concerned that their withdrawal symptoms are not always recognised.^18^ A lack of clinical input when patients are seeking repeat prescriptions exacerbates the issue.^2^ Survey studies indicate that withdrawal experiences can be diverse, including physical and emotional changes.^9^, ^19^ These experiences, along with barriers to withdrawal, contribute to some patients’ reluctance to discontinue their medication. Barriers to discontinuation include fear of relapse, withdrawal symptoms, and inadequate medication management.^11^ Patient interviews highlight fear as the most significant barrier, as patients worry about depressive relapse.^14^, ^20^ A thematic synthesis of patient perspectives showed that there were many barriers and facilitators to stopping antidepressants, therefore addressing these concerns is likely to necessitate in‐depth discussions between patients and their GPs.^21^ These crucial conversations are more likely to occur when GPs initiate the topic of discontinuation. The patient–GP relationship plays a crucial role in patients’ decisions to continue or discontinue antidepressants.^12^

### 1.1 Study aims

While important for patient outcomes, the predominant focus on physical withdrawal symptoms and barriers to withdrawal in the literature means that the impact of antidepressant withdrawal on other life domains such as mood, social interactions, or cognitive processes have not been examined in depth, using qualitative methods. Gaining insight into changes in these domains would greatly benefit clinicians aiming to guide patients through withdrawal safely and to give them a sense of what to expect. Consequently, this study aims to investigate patients’ lived experiences of antidepressant withdrawal, to gain a richer detailed understanding, across various life domains beyond physical symptoms, encompassing cognitive, social, and emotional functioning.

## 2 METHOD

### 2.1 Recruitment

We aimed to recruit up to 20 participants based on previous study guidelines for semi‐structured interviews analysed using thematic analysis.^22^, ^23^ The study was advertised on social media (recruitment poster on the first author's personal Twitter page), around campus at the University of Bath, and on the student Research Participation Scheme. Eligibility criteria were being aged between 18 and 65, being diagnosed with depression in the past (because some people take antidepressants for other conditions such as eating disorders), and having taken a SSRI antidepressant for depression. Crucially, participants’ withdrawal attempts had to have lasted at least a month. Participants were excluded if their withdrawal attempt had occurred >1 year ago (to limit recall and memory biases) and if they had taken their antidepressant medication for <6 months. Participants were either taking antidepressants at the time of the interview or had completely withdrawn (none were currently withdrawing).

### 2.2 Data collection

Participants were sent an information sheet at least 24 h before attending an online appointment. During this session, the researcher explained the study's key features, checked eligibility criteria, and answered any questions. Informed consent was obtained, and participants completed questionnaires assessing demographic characteristics, depression history, and current antidepressant use. Participants also completed the Patient Health Questionnaire‐9 (PHQ‐9) to measure current depressive symptoms.^24^

Participants were then asked to discuss their most recent withdrawal attempt. A topic guide was used to guide the semi‐structured interviews (see Supplementary Materials). Interviews lasted 40 min on average (range: 30–45 min). Participants were entered into a prize draw to win a £50 gift voucher or, if a Psychology student, were awarded course credit.

### 2.3 Sample

In total, 29 individuals expressed an interest in taking part; 6 were ineligible and 3 ultimately declined to participate. Thus, we conducted in‐depth semi‐structured interviews with 20 antidepressant users who had attempted to withdraw in the past year. All individual interviews took place via video call between June 2022 and February 2023. Of the 20 participants interviewed, 15 (75%) were female, and their mean age was 23.5 years (ranging from 18 to 42 years old). The majority of the participants (17 out of 20) were students. Thirteen participants had a PHQ‐9 score of ≥10 suggesting moderate to severe depression up to a year after their withdrawal attempt. Eleven participants were taking antidepressants at the time (these were participants 1–4, 10–12, and 14–17) and 70% of the sample (*n* = 14) reported tapering their medication when withdrawing (see Table 1 for further information).

**Table 1:** Demographic and clinical characteristics of the sample.

*N*	20
Age (years), mean (SD)	23.5 (7)
Gender, *N* (%)	
Female	15 (75)
Male	5 (25)
Ethnicity, *N* (%)	
Caucasian	16 (80)
Black	1 (5)
Asian	2 (10)
Mixed	1 (5)
Occupation, *N* (%)	
Student	17 (85)
Employed	2 (10)
Unemployed	1 (5)
Relationship, *N* (%)	
Single	5 (25)
In a relationship (not cohabiting)	11 (55)
Cohabiting	3 (15)
Married/in a civil partnership	1 (5)
PHQ‐9 score, mean (SD)	11.8 (7)
PHQ‐9 score, categories, *N* (%)	
No depression (<5)	4 (20)
Mild (5–9)	3 (15)
Moderate (10–14)	5 (25)
Moderately severe (15–19)	5 (25)
Severe (20 or more)	3 (15)
Tapered withdrawal, *N* (%)	
Yes	14 (70)
No	6 (30)
Depression status (self‐report), *N* (%)	
Yes, currently	13 (65)
In the past, not currently	7 (35)
Recurrence of depression after withdrawal (self‐report), *N* (%)	
Yes	14 (70)
No	6 (30)
Currently taking SSRI antidepressants, *N* (%)	
Yes	11 (55)
No	9 (45)
Antidepressant medication being taken at time of withdrawal, *N* (%)	
Sertraline	11 (55)
Citalopram	2 (10)
Fluoxetine	6 (30)
Paroxetine	1 (5)

Abbreviations: PHQ‐9, Patient Health Questionnaire‐9; SSRI, Selective Serotonin Reuptake Inhibitor.

### 2.4 Data analysis

All interviews were video recorded, transcribed verbatim, and then the data were anonymised. We used inductive reflexive thematic analysis.^25^, ^26^, ^27^ Thematic analysis provides a flexible framework for generating common themes and meanings across participants while allowing for individual meanings to be expressed.^27^ It is also not tied to a particular epistemological or theoretical perspective.^28^ The lead researcher implemented the six‐phase process outlined by Braun and Clarke.^25^ He familiarised himself with the data, making initial notes in the margins. Subsequently, both semantic and latent codes were generated. Themes were then derived after reviewing how different codes may be combined according to shared meanings. Candidate themes were then further refined and reviewed with the wider team. Thematic maps were developed to clarify the relationships between sub‐themes and themes and identify the final themes. Quotations were chosen to represent each theme and subtheme.

## 3 RESULTS

**Figure 1:**
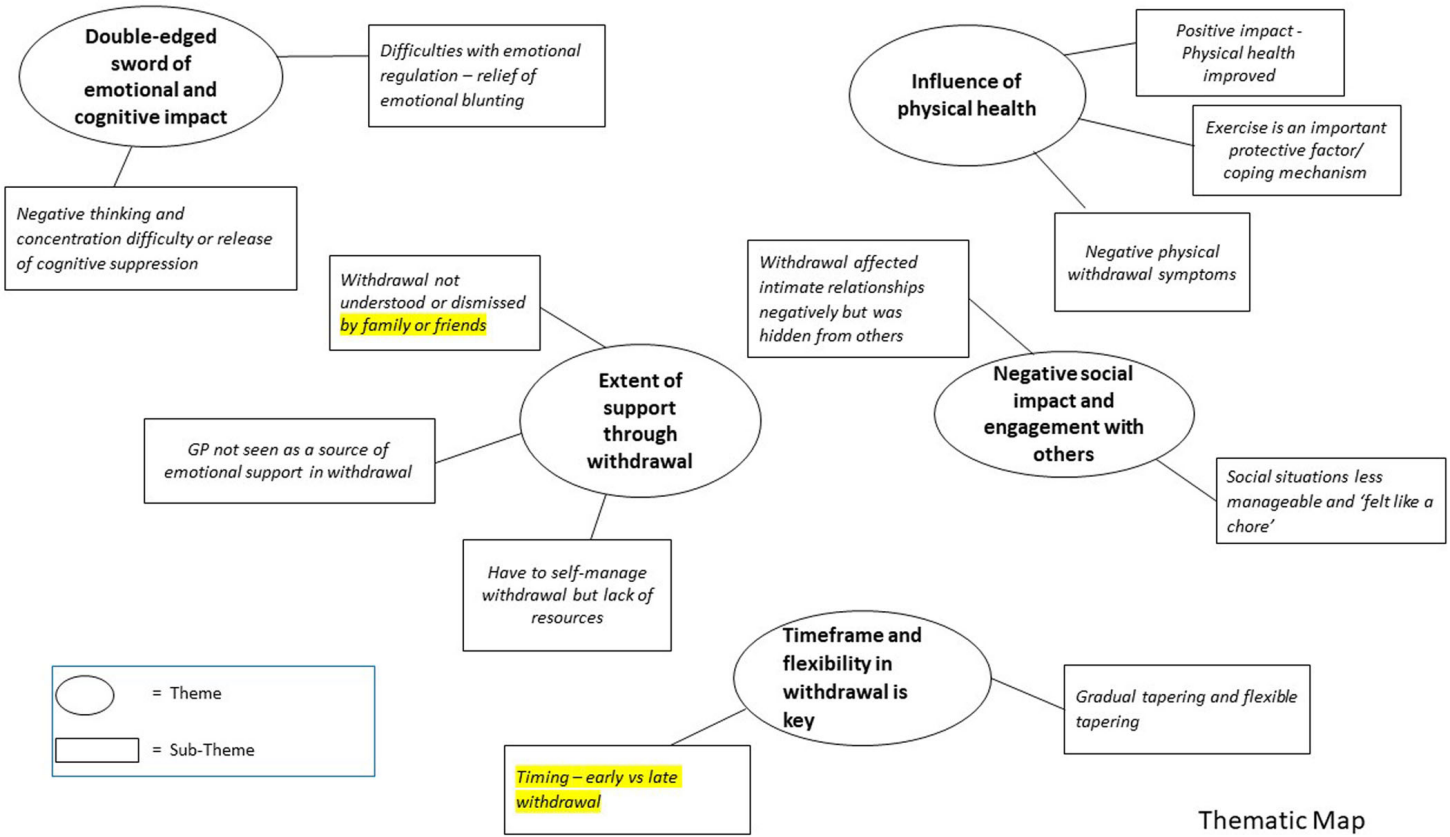
Thematic map.

Participants reported several reasons for wanting to withdraw from their SSRIs. These included the negative side effects while being on them, in particular sexual dysfunction and lack of sex drive, with many commenting that their personal relationships influenced their decision to come off. Others wanted to see if they could manage without them and did not want to be reliant on or felt uncomfortable about being on antidepressants in the long term. A few participants explained that they did not want to be an 'emotionless robot'. Finally, others reported experiencing difficulties in receiving and collecting prescriptions which prompted them to come off their medication after not taking their tablets for a few days and realising they might be able to manage without them. We identified five overarching themes (see Figure 1) which are described below with their respective subthemes.

### 3.1 Theme 1: ‘Double‐edged sword’ of emotional and cognitive impact

Participants described the emotional and cognitive impact of withdrawal as a double‐edged sword. They felt that the release from emotional blunting was sometimes difficult to manage but also appreciated being able to feel emotions again in a normal way. Similarly, participants reported increases in both positive and negative thoughts and concentration difficulties.

#### 3.1.1 Difficulties with emotional regulation: relief of emotional blunting

More than half of participants said that withdrawal relieved their emotional blunting. Participants experienced more (or heightened) emotions:

I think I've felt more emotional, during withdrawal and after. So, if we watch a film or if I'm reading a book, or in conversation with somebody, it's been feeling those emotions much more deeply than I did when I was on antidepressants. When you're taking the medication, the emotions have been dialled down. When withdrawing the emotions get heightened. (P5)

However, although participants reported richer emotional experiences, the sudden return of intense emotions could be overwhelming and initially difficult to manage:

It was a bit unstable, and things would upset me more easily than before. (P6)

I was watching something on TV innocuous, it might even have been Countryfile… and I got a bit teary watching it. It was something about a lamb, and I thought, well, it's not the kind of thing that would usually make me emotional. I think I definitely did feel a little bit more delicate during the tapering period and then later also. (P5)

I think it was just because I was feeling very neutral before and then it was a bit like up and down. I think in the first few days and weeks it was very emotional. Like I cried a lot. I was very impulsive as well’.(P11)

The return of emotions could be described as a double‐edged sword as both positive and negative emotions were amplified:

So, I felt like when I was happy, I was really happy but then that's the same for being really sad. (P16)

I got snappier more easily and prone to anger outbursts over trivial things. (P17)

And I guess that is the difficulty, that's the kind of the balance, you get back some of the emotions in brighter colours but also the things you found difficult will be a bit heightened…. (P5)

#### 3.1.2 Negative thinking and concentration difficulties or release of cognitive suppression

Similarly, a few participants felt that their cognitive suppression was released (thoughts and memories that had seemingly been suppressed were now coming back). Negative thinking and concentration difficulties were common, but some participants experienced a noticeable increase in positive thoughts:

Old memories came flooding back during withdrawal that have been suffocated for years…my thinking has been suppressed for such a long time and then the coming off the drug is almost like my body is recalibrating how to handle… any stimulation, and that includes thoughts itself. (P2)

I'd say I was thinking very negatively. So, for example, with my PhD work I had this complete crisis of confidence and thought that everyone else didn't know what I was doing. (P1)

I was probably having more thoughts. I think more positive, to be honest, than when I was on them. But then it did start to deteriorate. I think it sort of went up then down that's why I did go back on them. (P8)

A couple of participants also experienced a recurrence of overthinking; their thoughts spiralled, leading to mood deterioration and catastrophising:

I had a very negative thought process and everything that happened just built up and built up in my head and then became an issue for me. For example, I had a really minor argument with one of my friends. I was sitting there thinking we would never speak again and would not be friends. But literally, a few weeks later, I spoke to them, and they hadn't got an issue with me. (P10)

### 3.2 Theme 2: Negative social impact and engagement with others

Participants reported difficulties in close relationships, but withdrawal was hidden from others to maintain appearances. Participants also reported feeling socially detached and less empathetic during the withdrawal process.

#### 3.2.1 Withdrawal affected intimate relationships negatively

More than half of the participants felt that withdrawal negatively affected their intimate or close relationships. It was often close relations that had to deal with the participants’ emotion regulation difficulties:

My dad always got the brunt of it. Someone could say something to me, and I'd just snap and my mum, my family, my friends, they know it's not me. I can't help it. (P17)

Participants also felt that family members became more wary of their behaviour:

I was so sensitive and irritable to things, it became scarier for them to deal with me, and they were just treading on light waters. I think they were just scared to do anything that would upset me. (P20)

Some participants felt that their relatives preferred them to go back on antidepressants for reassurance:

It makes her feel as if there's like a safety bubble around me. (P17)

I think they are more relieved that I'm back on them it probably gives them some sort of peace of mind that I'm helping myself. (P11)

However, they also reported keeping withdrawal hidden from non‐intimate others:

Well, I don't really reveal much about what I'm going through with my friends and my social circle (P13)

I did kind of push myself to go out quite a bit because I didn't want anyone to know, and you know keeping up appearances. (P14)

#### 3.2.2 Social situations less manageable and ‘felt like a chore’

Following withdrawal, some participants reported that social situations felt laborious:

My friend got married and I was her celebrant for her wedding, the whole thing just felt like a chore. (P1)

Going out with my friends was a real pain. It was something l had to drag myself to do, and I wasn't enjoying it (P17).

Participants highlighted challenges in maintaining relationships and attending social events:

I realised that I needed more effort in the maintenance of my relationships like I need to push myself more. (P13)

Participants also reported feeling detached and less ‘present’ in relationships:

It definitely affected our relationship because I was down and very preoccupied. And I think that not being really present was probably the thing that characterised all of my relationships at the time. (P1)

A few participants reported becoming socially withdrawn:

Umm so when I just came off, I was definitely like more interested in hanging out with everyone still, but then as time went on, I think I got more and more reclusive. (P10)

### 3.3 Theme 3: Influence on physical health

A small number of participants reported a positive impact of withdrawal on their physical health. Exercise was identified as an important protective factor and coping mechanism. However, participants also reported a range of negative physical symptoms.

#### 3.3.1 Positive impact of withdrawal on physical health

Some participants reported being *‘*fitter, healthier and losing weight’ (P4). Being off antidepressants also helped them to better regulate their appetite:

I've noticed that every time I've tried to come off, I've lost weight and every time I've gone back on it, my appetite never feels fulfilled. I don't ever feel sated when I'm on it. Nowadays, I'm a lot better and I feel full when I eat a meal. (P2)

#### 3.3.2 Exercise is an important protective factor/coping mechanism

A few participants stated that exercise helped them manage negative withdrawal effects:

I was more active and that gave me a buffer for so long and that's why I was able to be off them for around 4 or five months…the first thing for me that goes when my mood is low is my ability to exercise and socialise. (P4)

Participants reported that they felt more willing to exercise, and this helped them to feel mentally better:

Because that drive to actually get out and exercise for me is what makes the difference. (P4)

I thought exercise was really good for mental health, So, I was trying to find ways that would make me happier. (P8)

#### 3.3.3 Negative physical withdrawal symptoms

All of the participants reported experiencing physical side effects during withdrawal, including tiredness and nausea, sleep problems including nightmares, night sweats and vivid dreams, diarrhoea, akathisia (inability to remain still; *‘*I feel like a wild animal stuck in barbed wire’), vomiting, brain zaps and electrical sensations, sweating, ringing in ears, headaches, dizziness, and joint pain.

Physical side effects were seen to impact their general quality of life but also affected their academic performance and social life:

It really affected things like doing essays, so I had to get like so many extensions. (P7)

I would rarely go out because I knew I'd get tired immediately so I would just stay inside. (P7)

### 3.4 Theme 4: Extent of support during withdrawal

Participants reported mixed experiences of support from family members, and their mental health literacy was identified as important. GPs were primarily seen as prescribers of medication even though participants were hoping for more guidance, resources, and emotional support from them.

#### 3.4.1 Withdrawal not understood or dismissed by family or friends

Around half of the participants reported struggling to communicate the challenges of withdrawal to family and friends, sometimes leading to conflict:

My mum just didn't believe me when I told her about the withdrawal effects. She was of the view that I should stop cold turkey and just toughen up. (P2)

This led to feelings of being dismissed and not understood, sometimes contributing to relationship breakdowns:

Well, I just lost a lot of my friends. And the friends I did have, I wasn't that keen on as they didn't really understand my withdrawal problems. (P20)

Participants who described receiving good support during withdrawal commented this was perhaps because their family members were mental health literate or had gone through similar experiences:

They were very supportive. My mum and my sister. I think my sister is on Sertraline and my mum has been on and off antidepressants for the past 20 years. So, they understand it. (P18)

I've got a partner who I live with…he supported me a lot and was quite mental health literate, so understands. (P1)

#### 3.4.2 GP not seen as a source of emotional support

Participants felt that although GPs were overall ‘*kind and supportive’ (P1)*, they had minimal involvement during withdrawal. This was seen as the way the system works:

So they're kind of mostly the provider of the pills and sick notes, which isn't diminishing GPs it's just the way it seems to work. (P1)

Participants also reported that there seemed to be a lack of awareness about withdrawal effects and insufficient follow‐up appointments:

So I've seen 6 doctors and I would say only one out of six recognised that antidepressants could cause withdrawal effects. (P2)

I kind of feel I got lost in the system with taking mine, so I never really had a lot of check‐ups. I want my GP to be more active and check in. (P11)

Participants expressed a desire for GPs to provide more advice about managing side effects and on‐going emotional support:

They didn't have any advice for managing the negative side effects of the withdrawal. They just said, if it starts getting too much then you might want to go back on them. (P8)

I started some mentoring through the university. I talked to my supervisors and friends and just wonder whether having someone to talk to you throughout it, having like a therapy check in, might be helpful. (P1)

#### 3.4.3 Have to self‐manage withdrawal but lack of resources

The limited role and involvement of GPs during withdrawal meant that participants felt they were often left to their own devices to manage side effects, but few self‐help resources were available from their healthcare providers:

I'm self‐taught in this stuff and I'm on shaky ground. I'd love to get my advice from the Doctor, not the Internet. (P2)

Most of the stuff I found out when I was going through withdrawal was just through Google and that's never the most reputable thing. But I was not explained stuff in great detail. (P18)

However, one participant noted there ‘seems to be a bit more coverage about this kind of thing now and it's getting better’ (P2).

### 3.5 Theme 5: Timeframe and flexibility in withdrawal is key

Participants perceived differences between early and late withdrawal and advised timing withdrawal attempts for less busy periods of life. Many participants viewed tapering positively.

#### 3.5.1 Timing ‐ early versus late withdrawal

Timing was an underlying theme that was present across all the other themes. For example, physical withdrawal symptoms often started immediately after reducing the dose:

…it's then the next couple of days where you start getting like side effects and stuff. (P16)

but could also appear or worsen in the later stages of withdrawal:

I don't remember the ringing in my ear being that bad at the beginning. (P3)

Some participants reported positive experiences of withdrawal early on while others faced challenges. Conversely, some participants reported that later stages of withdrawal were more difficult.

Experiences at beginning and end of withdrawal were quite different’. (P5)

A few participants emphasised the importance of timing withdrawal attempts for non‐stressful periods of life:

…should be timed for when you are less busy in life as psychological effects can be difficult to manage. (P5)

They also emphasised the need for allowing things ‘…to settle before you can accurately evaluate whether you're sadder or not, so I would say like stick for a couple of weeks with tapering’ (P6).

#### 3.5.2 Gradual and flexible tapering

Participants generally viewed tapering positively and believed people should seek GP advice before withdrawing:

If tapering is planned carefully and properly then withdrawal symptoms can be avoided. (P2)

If someone was thinking about coming off antidepressants I would definitely say to taper and definitely to speak to their GP about it. (P5)

Those that did not stick with their tapering plan or withdrew ‘cold turkey’ wished they had not done so in hindsight:

I started off by tapering then I forgot to take it for about 4 or five days and then thought I could just go cold turkey and it didn't work due to the side effects. (P16)

Withdrawal was definitely a mistake without supervision. (P10).

Participants stated that if tapering was flexible, managing their withdrawal would be easier:

I kind of have to listen to my body and I changed my dose accordingly and kind of played it by ear which I don't know how good an idea it was, but it got me through it. (P19).

## 4 DISCUSSION

This study provides new insights into patients’ lived experiences of withdrawing from SSRI antidepressants, with several themes emerging from the analysis that have received little attention in previous qualitative research. These include the marked impact of antidepressant withdrawal on patients’ emotional, cognitive, and social functioning, and also the complexity of such impacts, with both positive and negative effects being reported across these domains. Some patients welcomed the restoration of normal emotions or release from emotional blunting and cognitive/memory suppression, while others found the intensity of the positive and negative emotions, they experienced overwhelming. Social situations were described as less enjoyable, and participants reported feeling detached and less empathetic. Some participants experienced positive physical health effects (including weight loss) and felt that exercise served as a crucial protective factor and coping mechanism. However, all participants experienced negative physical withdrawal symptoms at some stage. Participants frequently self‐managed their withdrawal due to limited GP involvement and reported insufficient resources or evidence‐based guidance. They expressed a desire for more emotional support from GPs and stressed the importance of flexible tapering. Timing was generated as an important theme that spanned all domains of functioning—with some patients finding the early stages of withdrawal (days or weeks) most challenging, while others struggled more in the later stages (months in).

### 4.1 Comparisons with existing literature

Previous research has shown that withdrawal from SSRIs can involve a challenging journey of tapering and long‐lasting physical symptoms. Many SSRI users will experience withdrawal symptoms.^29^ Our study aligns with this, highlighting that physical side effects were experienced by all of the participants, and while they varied in severity and duration, they typically occurred early in the withdrawal process.  However, participants also emphasised the impact of withdrawal on their emotions, social interactions, and thinking patterns.

Notably, our study revealed positive effects of withdrawal on physical health, with some participants reporting improved physical health and a greater desire to exercise. Exercise has been recognised as a potential adjunctive treatment for depression.^30^, ^31^ In the context of antidepressant withdrawal, exercise may play a role in managing the process. However, it is important to acknowledge that the effects of exercise on depression and antidepressant withdrawal can vary among individuals, and the relationship between exercise and depression is not clear‐cut.^32^

Our study also highlighted that withdrawal may relieve the emotional blunting that is frequently experienced by antidepressant users. Emotional blunting is a common reason for patients choosing to discontinue antidepressants,^33^ yet little is known about the impact of antidepressant withdrawal on such blunting.^34^ Our study suggests that the release from emotional blunting can be difficult to manage, although despite this many participants welcomed the restoration of normal emotions. Previous research has found links between depressive symptoms and negative social interactions,^35^, ^36^ potentially explaining our findings. However, participants also expressed that the withdrawal exper in itself made them feel detached and less empathetic overall, regardless of physical withdrawal symptoms. Participants reported hiding the fact that they were withdrawing from non‐intimate others, reflecting the negative social perceptions surrounding mental health problems and antidepressant use.^37^ These negative perceptions may contribute to feelings of isolation and prevent individuals from receiving the support they need during the withdrawal process.

The subtheme of self‐managed withdrawal with limited resources aligns with other research, with one prior survey study (*n* = 342) identifying subthemes related to insufficient information and support.^9^ Research also indicates a discrepancy between healthcare professionals’ claims that patients are informed about withdrawal symptoms and patient reports.^38^ This was evident in a recent large online survey (*n* > 1000) which revealed that less than 5% of patients reported being told anything about antidepressant withdrawal effects by their prescribers.^39^ Our findings are also supported by another recent qualitative study, where participants highlighted the need for more support from healthcare professionals, a greater focus on non‐pharmacological approaches in primary care to address unnecessary antidepressant use, and increased awareness of the withdrawal process.^40^ Importantly, these concerns are further reflected in national guidelines where in a 2019 report Public Health England recognised the significant challenges individuals face when withdrawing from antidepressants and called for the implementation of specialised support services and a helpline for individuals attempting to withdraw from antidepressants,^41^ a need highlighted by patients as being currently lacking.^42^, ^43^ Previous research has also shown that social media (e.g., online support communities) often fills a void left by healthcare services with the most common reason for people joining these groups being perceived lack of support from clinicians.^44^ These findings underscore the need for enhanced support and understanding from healthcare professionals. A recent qualitative study of Australian GPs’ views on antidepressant discontinuation demonstrated that GPs are aware of the need to consider patients’ social relationships and wider context (employment amongst others) when planning withdrawal.^45^ However our study and the results of large survey studies^39^ suggest that GPs in England may not be as aware of these issues.

### 4.2 Strengths and limitations

Through semi‐structured interviews, we gained a richer perspective on lived experiences of withdrawal and its impact on participants’ quality of life. Participants were able to raise issues around withdrawal that were important to them. Our study focused on the less well studied aspects of withdrawal, including social, emotional, and cognitive impacts, in addition to physical symptoms. These domains impact each other and need to be considered by GPs when managing withdrawal in conjunction with patients. To mitigate recall biases, participants reported specifically on withdrawal attempts within the past year.

However, our sample consisted primarily of (female) university students, potentially limiting the range of experiences. Their duration of antidepressant use may have been shorter than many antidepressant users in the general population, and the withdrawal attempt they described may have been their first (although this information was not collected during the study). Future research should involve larger and more diverse samples in terms of age, occupation, ethnicity, duration of antidepressant use, and number of previous withdrawal attempts. Furthermore, participants who volunteered to take part (including those recruited from social media adverts) may have had stronger opinions or different experiences of antidepressant withdrawal compared to antidepressant users from the general population. Such self‐selection biases could limit the sample's representativeness. Additionally, we cannot be sure that the specific experiences reported in this paper were entirely due to withdrawal, as participants’ experiences may have been influenced by other factors unrelated to withdrawal, such as negative life events (e.g., bereavement). Given that many of the participants were depressed at the time of the interview, based on their PHQ‐9 scores, it should also be noted that there are challenges in distinguishing between withdrawal effects/symptoms and depressive relapse in this context.

In our study, 65% of the participants met the clinical cut‐off for depression at the time of interview (PHQ‐9 score of ≥10), suggesting at least moderate depression up to a year after attempting to withdraw from antidepressants which could have impacted on the experiences they reported. This potential relapse rate (we did not objectively measure relapse) resembles that observed in the wider population of antidepressant users and is comparable to a longitudinal study which found that 56% of those who withdrew relapsed over a 1‐year period.^46^ However, it is difficult to draw comparisons about relapse rates from our qualitative study to longitudinal observational studies that measured relapse over time. Another limitation of our study is that we did not collect detailed information on tapering plans and whether participants stuck to them, but this would be an important factor to examine in future studies.

### 4.3 Implications for practice and research

When discussing withdrawal with patients, it may be helpful for GPs to explain that they may experience challenges in the emotional, social, and cognitive domains, in addition to physical withdrawal symptoms that can persist for months. It is important to emphasise that withdrawal can be manageable if the tapering plan is flexible and the patient takes a cautious approach to withdrawal, monitoring how they feel and adapting their taper accordingly. GPs may consider communicating to their patients that engaging in physical exercise, maintaining a healthy lifestyle, and seeking social support may help mitigate the negative aspects of antidepressant withdrawal. GPs could advise their patients to inform others about what they are going through or seek out social support, because it can be a challenging time for them socially. Patients may find it helpful to talk to close family or friends about their plans to taper and what changes, for example mood swings, they might expect. It might also be advantageous to time their withdrawal attempts for less stressful or busy periods in their lives. However, this study also shows that some patients may find certain aspects of withdrawal positive, such as the reduction of emotional blunting and the return of positive thoughts and memories that had seemingly been suppressed during antidepressant treatment. Similar to withdrawal, antidepressant treatment also has a range of negative side effects. For example, several participants reported that antidepressant side effects related to sexual dysfunction were a key reason for discontinuing their medication. Indeed, some participants commented on the improvements in the physical, emotional, and cognitive domains in our study. Thus, GPs should make patients aware of the potential positive and negative aspects of the withdrawal experience and have an open and honest dialogue with them, bearing these points in mind, when reviewing their medication.

This study highlights some encouraging avenues for future research, such as investigating the role of GP support (participants expressed a desire for more support from GPs), fostering social support, and examining the potential protective role of exercise in the withdrawal experience. To delve deeper into these rich experiences, one area for future research might be to conduct a longitudinal qualitative study where patients are interviewed at different stages as they come off their SSRIs. This approach would enable researchers to compare the lived experiences of withdrawal before, during, and after the withdrawal process and examine whether patients stick to their tapering plans or not and how this impacts the antidepressant withdrawal experience. It would also aid in understanding potential variations in the time course of changes in these domains and assessing the critical factors contributing to a manageable withdrawal experience.

## AUTHOR CONTRIBUTIONS

**Raqeeb Mahmood**: Conceptualisation; investigation; writing—original draft; writing—review and editing; formal analysis; funding acquisition. **Vuokko Wallace**: Supervision; formal analysis; writing—review and editing. **Nicola Wiles**: Writing—review and editing. **David Kessler**: Writing—review and editing. **Katherine S. Button**: Conceptualisation; funding acquisition; writing—review and editing; supervision. **Graeme Fairchild**: Supervision; conceptualisation; funding acquisition; writing—review and editing; formal analysis.

## CONFLICT OF INTEREST STATEMENT

The authors declare no conflict of interest.

## ETHICS STATEMENT

Ethical approval for this study was granted by the University of Bath's Psychology Research Ethics Committee (Ethics code: 22‐028). Digital informed consent was obtained from all participants.

## Supporting information

Supporting information.

## Data Availability

The data underlying this article cannot be shared publicly to protect the privacy of the participants, beyond what is contained within the report. Further information can be requested from the corresponding author.
