# Supplementary material for: The lived experience of withdrawal from Selective Serotonin Reuptake Inhibitor (SSRI) antidepressants: A qualitative interview study
Source: Health Expect. 2024 Jan 9;27(1):e13966. doi: 10.1111/hex.13966 (PMC10774987; doi:10.1111/hex.13966)
Supplement: Supplementary file 1 — Supporting information. [file HEX-27-e13966-s001.docx]

**Supplementary 1 – Topic Guide of Questions**

*General guide of questions to navigate the semi-structured interview, follow-up questions and new questions should be asked, depending on the interviewee's responses.*

**Antidepressant withdrawal experience**

1. Why did you stop taking your antidepressant and how did you come to this decision (i.e., did you withdraw under clinical supervision)?
2. What was it like coming off the medication initially (first few days or weeks) and over the longer term (weeks or months, if applicable)?
3. Can you tell me about your relationships with your family and friends while you were withdrawing from antidepressants?
4. What was your social life like (in terms of attending events, for example) during the withdrawal process?
5. Can you tell me whether you noticed any change in your thinking during the withdrawal process? Can you give examples?
6. Can you tell me whether you noticed any change in your mood during the withdrawal process? Can you give examples?
7. Can you tell me whether you noticed any change in your physical health during the withdrawal process.
